# Increasing incidence of non-HBV- and non-HCV-related hepatocellular carcinoma: single-institution 20-year study

**DOI:** 10.1186/s12876-021-01884-5

**Published:** 2021-07-31

**Authors:** Yuko Nagaoki, Hideyuki Hyogo, Yuwa Ando, Yumi Kosaka, Shinsuke Uchikawa, Yuno Nishida, Yuji Teraoka, Kei Morio, Hatsue Fujino, Atsushi Ono, Takashi Nakahara, Eisuke Murakami, Masami Yamauchi, Wataru Okamoto, Tomokazu Kawaoka, Masataka Tsuge, Akira Hiramatsu, Daiki Miki, Michio Imamura, Shoichi Takahashi, Kazuaki Chayama, Hiroshi Aikata

**Affiliations:** 1 Department of Gastroenterology and Metabolism, Graduate School of Biomedical and Health Science, Hiroshima University, 1-2-3 Kasumi, Minami-ku, Hiroshima, 734-8551 Japan; 2 Research Center for Hepatology and Gastroenterology, Hiroshima University, Hiroshima, Japan; 3 Cancer Treatment Center, Hiroshima University Hospital, Hiroshima, Japan; 4 Natural Science Center for Basic Research and Development, Hiroshima University, Hiroshima, Japan; 5 Institute of Physical and Chemical Research (RIKEN) Center for Integrative Medical Sciences, Yokohama, Japan; 6 Collaborative Research Laboratory of Medical Innovation, Hiroshima University, Hiroshima, Japan

**Keywords:** HCC, NBNC-HCC, NASH, Cryptogenic

## Abstract

**Background:**

We previously reported on the trends in the etiologies of hepatocellular carcinoma (HCC) diagnosed in patients between 1995 and 2009. The aims of our updated study were to evaluate the incidence, nonhepatitis B and nonhepatitis C viral (NBNC) etiologies, and clinical characteristics of HCCs occurring in patients between 1992 and 2018.

**Methods:**

The study enrolled 2171 consecutive patients with HCC between 1992 and 2018. Their medical records were reviewed. The patients were divided into two groups, patients with early diagnoses from 1992 to 2009 and those with late diagnoses from 2010 to 2018.

**Results:**

NBNC-HCC occurred in 514 patients (23.6%). The percentage of patients with HCC who had NBNC-HCC increased from 26.5% in 2009 to 46.3% in 2018. Patients with NBNC-HCC were older (median ages from 67 to 73 years). Type 2 diabetes mellitus (48.5–60.3%: *P* = 0.008), hypertension (48.5–57.4%: *P* = 0.047), and hyperlipidemia (39.2–53.8%: *P* = 0.001) increased significantly in recent years. The median FIB-4 index decreased (4.37–3.61: *P* = 0.026) and the median platelet count increased (15.1–17.9 × 10^4^/μL: *P* = 0.013). Among the 514 patients with NBNC-HCC, 194 underwent hepatic resection for nonalcoholic steatohepatitis (NASH) (15%), alcoholic liver disease (ALD) (29%), and cryptogenic hepatitis (56%). Cirrhosis was detected in 72%, 39%, and 16% of patients with NASH, ALD, and cryptogenic hepatitis, respectively. The prevalence of cirrhosis in patients with NASH was significantly higher than the prevalence of cirrhosis in the other groups (*P* < 0.001). Overall, 70% of the non-malignant liver tissue of patients with NBNC-HCC was not involved with cirrhosis. On the other hand, the median FIB-4 index in patients with cryptogenic HCC was 2.56, which was a significantly lower value than those values in the other groups of patients. The FIB-4 index considered as one of useful screening of HCC.

**Conclusions:**

The prevalence of NBNC-HCC has increased rapidly even in a regional university hospital. Metabolic syndrome may be an important risk factor for HCC. HCC was also found in patients with non-cirrhotic livers. The FIB-4 index may be a useful screening method for HCC in patients with NBNC.

## Background

Hepatocellular carcinoma (HCC) is one of the most common malignant tumors worldwide. It ranks fifth in men and sixth in women as a cause of death from malignancies in Japan [1]. HCC accounts for 93.3% of primary liver cancers [2]. Up until now, hepatitis C virus (HCV) and hepatitis B virus (HBV) have contributed to the development of primary liver cancer, especially HCC. Cases of HCV-related and HBV-related HCC have accounted for 67% and 16%, respectively, of the vast majority of such cases worldwide [3].

However, efforts to control HBV and HCV infections have led to decreases in the mortality rates in Asia and southern Europe [4]. The long-term suppression of active chronic HBV infection by nucleoside/nucleotide analogs may reduce the incidence of HBV-related HCC [5, 6], and the eradication of HCV by antiviral therapy can reduce the occurrence of HCV-related HCC [7].

According to our previous study [8], 67% and 17% of HCCs were related to HCV–HCC and HBV–HCC, respectively. Fifteen percent of those cases were negative for antibody to HCV (HCVAb) and hepatitis B surface antigen (HBsAg) (NBNC-HCC). The incidence of HCV-related HCC has gradually decreased in recent years, whereas the incidence of NBNC-HCC has gradually increased from 10% in 1995 to 19% in 2009 [8]. Furthermore, Tateishi et al. reported that the incidence of NBNC-HCC has increased from 10% in 1991 to 32.5% in 2015 [9, 10].

Nonalcoholic fatty liver disease (NAFLD) and alcoholic liver disease (ALD) have been reported to be risk factors of NBNC-HCC. Fibrosis develops in 20–40% of alcoholics, and 10–20% of patients with fibrosis eventually progress to cirrhosis [11]. Approximately 3% of patients with fibrosis progress to cirrhosis annually [12], and HCC is diagnosed in 1–2% of patients with cirrhosis every year [13].

NAFLD and nonalcoholic fatty steatohepatitis (NASH) (both hepatic manifestations of obesity) and T2DM have been clearly linked to the progression of liver fibrosis to cirrhosis and risk of HCC in several recent studies [14–16]. Additional evidence has also suggested that obesity and T2DM are risk factors of HCC [17–20]. In Japan, overweight and obese patients have a 1.74 relative risk for HCC compared with normal and under-weight individuals [21]. Patients with T2DM have a two- to fourfold greater risk of HCC than nondiabetic control individuals [22–25].

Insulin resistance (IR) is associated with metabolic syndrome. T2DM and NAFLD leads to the increased release of free fatty acids from adipocytes and the release of multiple pro-inflammatory cytokines, including tumor necrosis factor alpha (TNF-α), interleukin-6 (IL-6), leptin, and resistin; and decreased secretion of adiponectin. Obesity is characterized by a low-grade chronic inflammatory response that is associated with increased in HCC [14, 26–29]. T2DM, obesity, and the metabolic syndrome have been growing in prevalence and are independent risk factors for the development of HCC.

Whereas many studies have reported on the risks of NBNC-HCC, there have been few published reports on the histopathological characteristics of liver specimens obtained by hepatectomy from patients with HCC. Several studies have reported that patients with NASH may progress to cirrhosis, and advanced fibrosis is an important risk factor for HCC [16, 30–32] and the prevalence of cirrhosis in the ALD-HCC was significantly higher, too [11, 17, 33–36]. NASH and ALD have now become the leading indications for liver transplantation such as HCV and HBV.

The increasing numbers of cases with NBNC-HCC related to NAFLD, ALD, obesity, T2DM, and metabolic syndrome have recently become a serious problem in Japan. However, the clinical features of such patients with HCC, including those with NBNC-HCC, have not been fully elucidated in Japan. Therefore, because of the urgent need to prevent the development of HCC and to develop strategies for the management of NBNC-HCC, we investigated the histopathological characteristics of NBNC-HCC in liver specimens.

## Methods

### Patients

We retrospectively reviewed the data of 2171 consecutive patients with the diagnosis of primary HCC, who were seen at Hiroshima University Hospital from January 1992 to December 2018. All the study participants provided informed consent to be enrolled in the study, in accordance with the process approved by the ethical committee of Hiroshima University Hospital. The study conformed to the ethical guidelines of the Declaration of Helsinki.

### Laboratory evaluations

We collected detailed clinical data on the patients (age, gender, history of habitual alcohol consumption, height, weight, lifestyle-related diseases, and laboratory parameters).

Standard techniques were used to evaluate the biochemical data of each patient at the time of diagnosis of primary HCC. The fibrosis (FIB)-4 index was calculated and used as a surrogate marker of liver fibrosis [37]. The FIB-4 index = age (years) × aspartate aminotransferase (AST) level [IU/L]/(platelet count [10^9^/L] × (ALT [IU/L])^1/2^). The albumin-bilirubin (ALBI) grade was used as an assessment tool for hepatic functional reserve. The ALBI grade was based on serum albumin and total-bilirubin levels and calculated by the following formula: ALBI grade: (log_10_ bilirubin (μmol/L) × 0.66) + (albumin (g/L) × −0.085); ALBI grade was defined as score follows: ≤ −2.60 = Grade 1, > −2.60 to ≤ −1.39 = Grade 2, >−1.39 = Grade 3 [38].

Patients with HBV–HCC were patients positive for HBsAg and negative for HCVAb. Patients with HCV–HCC were patients positive for HCVAb and negative for HBsAg. Patients with NBNC-HCC were patients negative for both HBsAg and HCVAb.

A patient with alcoholic liver disease (ALD) was identified by an alcohol intake > than 60 g/day for 5 years [39]. The body mass index (BMI) was calculated as follows: body weight (kg) divided by the square of the height in meters (kg/m^2^). The estimated HbA1c (%) value was based on the equivalent value (%) from the national glycohemoglobin standardization program (NGSP) and was determined from the following formula: HbA1c (%) = HbA1c (Japan Diabetes Society [JDS]) (%) + 0.4%. It was based on the relational expression of HbA1c (JDS) (%) as measured by the previous Japanese standards for substance and measurement and HbA1c from the NGSP [40]. T2DM was diagnosed in patients with an elevated fasting plasma glucose level (FPG, ≥ 126 mg/dL), elevated HbA1c (NGSP) value (HbA1c, ≥ 6.5%), use of glucose-lowering agents, or a self-reported history of a clinical diagnosis of T2DM.

Hyperlipidemia was diagnosed when the patient was being treated with lipid-lowering medications or had elevated total cholesterol levels greater than 220 mg/dL and/or a triglyceride level greater than 150 mg/dL. Hypertension was diagnosed when the patient was on antihypertensive medications and/or had a resting recumbent blood pressure of ≥ 130/85 mmHg on at least 2 occasions.

### Histopathological evaluation

The histopathological status of patients with liver disease associated with HCC was determined by microscopic examination of noncancerous regions of the surgical or biopsy specimen stained by hematoxylin–eosin and Azan. All liver tissue specimens were evaluated by 2 senior pathologists who were unaware of the patients’ laboratory data and clinical courses. The histopathological diagnosis of NASH was confirmed based on the Brunt criteria [41, 42]. Steatosis was graded as follows: grade 1 (≥ 5% and < 33% of hepatocytes affected); grade 2 (33–66% of hepatocytes affected); or grade 3 (66% < of hepatocytes affected). Necroinflammation was graded 0 (absent) to 3 (1, occasional ballooned hepatocytes and no or very mild inflammation; 2, ballooning of hepatocytes and mild-to-moderate portal inflammation; and 3, intra-acinar inflammation and portal inflammation). Fibrosis was graded 0 (absent) to 4 (1, perisinusoidal/pericellular fibrosis; 2, periportal fibrosis; 3, bridging fibrosis; and 4, cirrhosis). Ballooning was graded 0 (none) to 2 (1, few ballooning cells; and 2, many cells/prominent ballooning) [41, 42].

The diagnosis of HCC was based on the hypervascular staining pattern of the arterial phase and the hypovascular staining pattern of the portal phase, and was confirmed by dynamic CT and magnetic resonance imaging, and/or angiography [43]. Tumors without enhancement upon imaging were diagnosed by fine needle biopsy. Clinical staging of HCCs (TNM classification) was performed according to the criteria of the Liver Cancer Study Group of Japan [44].

### Statistical analysis

Results are shown as median values or percentages. Continuous variables are reported as medians (deviation) and were compared by the Mann–Whitney U-test. Categorical variables were compared by the chi-squared or Fisher exact test, as appropriate. The Cochran Armitage trend test was used to evaluate increasing or decreasing trends. SPSS software, version 23.0 (IBM, Armonk, NY, USA) was used for statistical analysis. A *P* value < 0.05 was considered statistically significant.

## Results

### Trends in the etiologies of liver disease in patients with HCC

**Fig. 1:**
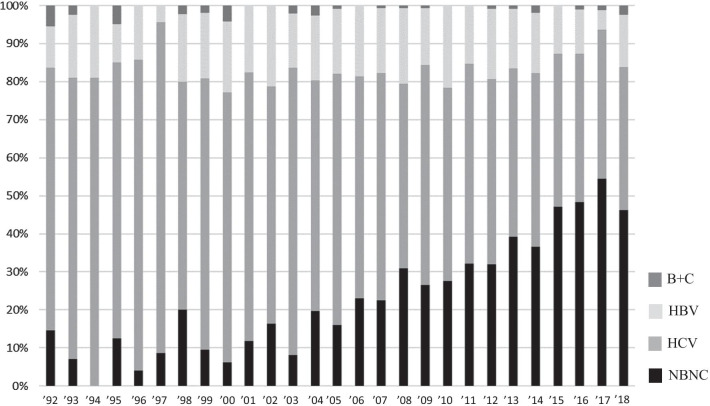
Trends in the causes of liver disease in patients with HCC over time. We show that distributions in the etiologies of liver disease in patients with HCC among 2171 patients from 1992 to 2018. The graph shows the actual number of NBNC-HCC.  : NBNC;  : HCV;  : HBV;  : B + C

Among 2171 patients with primary HCC from 1992 to 2018, HCV–HCC and HBV–HCC were found in 1271 (58.5%) and 361 (16.6%) patients, respectively (Fig. 1); 514 (23.6%) patients had NBNC-HCC and 25 (1.1%) patients were positive for both HBsAg and HCVAb (BC-HCC). To compare with the previous reports from 1995 to 2009, the study patients were divided according to 3 time periods.

The etiologies of HCC during the three periods were as follows. The percentages of patients who had HCV–HCC from 1992 to 2000, 2001 to 2009, and 2010 to 2018 were 74.0% (n = 311), 61.1% (n = 546), and 47.8% (n = 414), respectively. The percentages with HBV–HCC from 1992 to 2000, 2001 to 2009, and 2010 to 2018 were 14.2% (n = 60), 19.0% (n = 169), and 15.4% (n = 134), respectively. The percentages with NBNC-HCC from 1992 to 2000, 2001 to 2009, and 2010 to 2018 were 9% (n = 38), 18.6% (n = 166), and 35.7% (n = 310), respectively. The percentages with BC-HCC from 1992 to 2000, 2001 to 2009, and 2010 to 2018 were 2.4% (n = 10), 0.8% (n = 7), and 0.9% (n = 8), respectively. While the rate and number of patients with HCV–HCC tended to decrease, the rate and number of patients with NBNC-HCC increased significantly.

### Characteristics of patients with HCC based on etiology of liver diseases

The characteristics of the 2171 patients with HCC are shown in Table 1. Twenty-five patients with BC-HCC were excluded from the analysis because of the small number of patients. Of the 514 patients with NBNC-HCC, 417 were males and 97 were females. The median age was 72 years, 64.2% consumed elevated volumes of alcohol and 37.5% were positive for HBCAb. The median BMI was 24.3 kg/m^2^, 55.6% of patients had T2DM, 53.8% had hypertension, and 48.0% had hyperlipidemia. The median platelet count was 17.8 × 10^4^/μL, and the median FIB-4 index was 3.89. The ALBI grades were as follows: 231 grade 1 (n = 231), grade 2 (n = 242), and grade 3 (n = 41). The TNM stages of HCC were as follows: stage I (n = 54), stage II (n = 188), stage III (n = 141), and stage IV (n = 131).

**Table 1:** Characteristics of patients with HCC who were stratified according to etiology of their liver disease

	NBNC	HBV	HCV	*P* value	
	n = 514	n = 361	n = 1271	NBNC versus HBV	NBNC versus HCV
Age (years)	72 (21–90)	58 (31–80)	68 (28–84)	< 0.001	< 0.001
Sex (male/female)	417/97	294/67	841/430	NS	< 0.001
Alcohol consumption	330 (64.2%)	207 (57.3%)	562 (44.2%)	0.024	< 0.001
Antibody to hepatitis B core antigen	179 (34.8%)	361 (100%)	397 (31.2%)	< 0.001	< 0.001
Body mass index (kg/m^2^)	24.3 (14.3–41.2)	22.8 (14.4–31.7)	22.2 (14.8–33.6)	< 0.001	< 0.001
Diabetes mellitus	286 (55.6%)	73 (20.2%)	373 (29.3%)	< 0.001	< 0.001
Hypertension	277 (53.8%)	82 (22.7%)	527 (41.4%)	< 0.001	< 0.001
Hyperlipidemia	247 (48.0%)	105 (29.0%)	295 (23.2%)	< 0.001	< 0.001
ALT (IU/l)	47 (6–435)	55 (10–644)	51 (9–847)	< 0.001	< 0.001
Platelet counts (× 10^4^/μL, median)	17.8 (2.7–57.3)	15.6 (1.9–38.1)	11.7 (1.1–28.1)	< 0.001	< 0.001
FIB-4 index	3.89 (0.25–28.31)	4.93 (0.35–34.69)	6.63 (0.69–39.61)	< 0.001	< 0.001
ALBI grade 1/2/3	231/242/41	176/154/31	453/741/77	NS	< 0.001
Child–Pugh grade A/B/C	382/105/27	267/71/23	919/304/48	NS	NS
Antinuclear antibody	68 (13.2%)	46 (12.7%)	188 (14.9%)	NS	NS
Antimitochondrial antibodies	15 (2.9%)	3 (0.8%)	20 (1.6%)	0.032	NS
HCC stage^a^ (I/II/III/IV)	54/188/141/131	61/103/90/107	332/467/321/142	0.006	< 0.001

Continuous data are presented as medians and range, and categorical data are presented as numbers of patients

^a^Determined according to the criteria set by the Liver Cancer Study Group of Japan; *HBV* hepatitis B virus, *HCV* hepatitis C virus, *NBNC* negative for hepatitis B surface antigen and antibody to hepatitis C virus, *ALT* alanine aminotransferase, *FIB-4* fibrosis-4, *ALBI grade* albumin-bilirubin grade, *NS* not significant

Of the patients with HBV–HCC, 294 were males and 67 were females. The median age was 58 years, 57.3% consumed elevated volumes of alcohol, and 100% were positive for HBcAb. The median BMI was 22.8 kg/m^2^, and 20.2% of patients had T2DM, of whom 22.7% had hypertension, and 29.0% of those had hyperlipidemia. The median platelet count was 15.6 × 10^4^/μL and median FIB-4 index was 4.93. The ALBI grades were as follows: grade 1 (n = 176), grade 2 (n = 154), grade 3 (n = 31). The TNM stages of HCC were as follows: stage I (n = 61), stage II (n = 103), stage III (n = 90), and stage IV (n = 107).

Of the patients with HCV–HCC, 841 were males and 430 were females. The median age was 68 years, 44.2% consumed elevated daily volumes of alcohol, and 31.2% were positive for HBcAb. The median BMI was 22.2 kg/m^2^; and 29.3% of patients had T2DM, of whom 41.4% had hypertension and 23.2% had hyperlipidemia. The median platelet count was 11.7 × 10^4^/μL, and the median FIB-4 index was 6.63. The ALBI grades were as follows: grade 1 (n = 453), grade 2 (n = 741), grade 3 (n = 77). The TNM stages of HCC were as follows: stage I (n = 332), stage II (n = 467), stage III (n = 321), and stage IV (n = 142).

Patients with NBNC-HCC were significantly older than patients in the other groups (*P* < 0.001). The rates of complications from excessive alcohol consumption (*P* < 0.001), T2DM (*P* < 0.001), hypertension (*P* < 0.001), and hyperlipidemia (*P* < 0.001) in patients with NBNC-HCC were significantly higher than the rates of complications in other groups. The BMI and the platelet count of patients with NBNC-HCC were significantly higher than those values of the patients in the other groups (*P* < 0.001), and the FIB-4 index of the patients with NBNC-HCC was significantly lower than those values of the patients in other groups (*P* < 0.001). The TNM stages of HCC in the patients with NBNC-HCC were significantly more advanced than the stages of the patients in the other groups (vs. HBV–HCC, *P* = 0.006; vs. HCV–HCC, *P* < 0.001).

### Characteristics of patients with NBNC-HCC

Table 2 shows the characteristics of patients with NBNC-HCC stratified by the time periods 1992–2009 versus 2010–2018. Patients with NBNC-HCC from 2010 to 2018 were significantly older than those patients with NBNC-HCC from 1992 to 2009 (*P* < 0.001). As mentioned in the previous term, the incidence and numbers of patients with NBNC-HCC tended to increase; accordingly, the number of patients with metabolic syndrome also tended to increase.

**Table 2:** Characteristics of patients with NBNC-HCC stratified according to dates of study period

	1992–2009	2010–2018	*P* value
	n = 204	n = 310	2010–2018 versus 1992–2009
Age (years)	67 (21–85)	72 (22–90)	< 0.001
Sex (male/female)	163/41	254/56	NS
Alcohol consumption	127 (62.2%)	203 (65.4%)	NS
Antibody to hepatitis B core antigen	83 (40.6%)	96 (30.9%)	0.023
Body mass index (kg/m^2^)	23.8 (14.3–38.9)	23.9 (15.3–41.2)	NS
Diabetes mellitus	99 (48.5%)	187 (60.3%)	0.008
Hypertension	99 (48.5%)	178 (57.4%)	0.046
Hyperlipidemia	80 (39.2%)	167 (53.8%)	0.001
ALT (IU/L)	47 (8–435)	48 (6–388)	NS
Platelet counts (× 10^4^/μL, median)	15.1 (3.5–49.1)	17.9 (2.7–57.3)	0.013
FIB-4 index	4.37 (0.67–28.31)	3.61 (0.25–22.34)	0.026
ALBI grade 1/2/3	93/96/15	138/146/26	NS
Child–Pugh grade A/B/C	151/42/11	231/63/16	NS
Antinuclear antibody	23 (11.2%)	45 (14.5%)	NS
Antimitochondrial antibodies	9 (4.4%)	6 (1.9%)	NS

Continuous data are presented as medians and range, and categorical data are presented as numbers of patients

*NBNC* negative for hepatitis B surface antigen and antibody to hepatitis C virus, *ALT* alanine aminotransferase, *FIB-4* fibrosis-4; *ALBI grade* albumin-bilirubin grade, *NS* not significant

T2DM was found in 99 (48.5%) of 204 NBNC-HCC patients from 1992 to 2009 and 187 (60.3%) of 310 NBNC-HCC patients from 2010 to 2018 (*P* = 0.008). Hypertension was found in 99 (48.5%) NBNC-HCC patients from 1992 to 2009 and 178 (57.4%) patients from 2010 to 2018 (*P* = 0.046). Hyperlipidemia was found in 80 (39.2%) NBNC-HCC patients from 1992 to 2009 and 167 (53.8%) patients from 2010 to 2018 (*P* = 0.001). The numbers of patients with metabolic syndrome (T2DM, hypertension, hyperlipidemia) increased significantly. On the other hand, the median FIB-4 index of the NBNC-HCC patients from 1992 to 2009 and from 2010 to 2018 were 4.37 and 3.61, respectively; (*P* = 0.026) which was a significant decrease over the last 10 years. Furthermore, the median platelet counts increased from 1992 to 2009 to 2010 to 2018 (15.1 and 17.9, respectively; (*P* = 0.013).

### Comparison of FIB-4 index of patients with NBNC-HCC stratified by age ranges

**Fig. 2:**
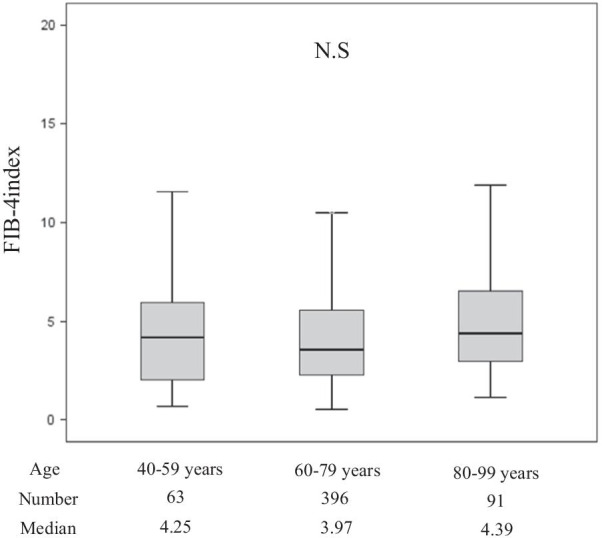
The FIB-4 index of patients with NBNC-HCC stratified by age. Lines within the boxes represent median values; the upper and lower lines of the boxes represent the 75th and 25th percentiles, respectively; the upper and lower bars outside the boxes represent the 90th and 10th percentiles, respectively. *NS* not significant

We assessed the FIB-4 indiex of patients with NBNC-HCC stratified by age ranges. The median FIB-4 index in patients aged from 20 to 39, 40 to 59, 60 to 79, and 80 to 99 years were 1.43, 4.25, 3.97, and 4.39, respectively. The differences between the FIB-4 index of patients stratified by ages starting at 40 years were not significant (Fig. 2).

### Clinical characteristics of patients with NBNC-HCC who underwent hepatic resection

Among the 514 patients with primary NBNC-HCC, we studied the clinical features of 194 patients who underwent hepatic resection (patients with autoimmune hepatitis or primary biliary cholangitis were excluded). We assessed the histological features of the hepatic tissue not involved with tumor. Those patients in whom neither NASH nor ALD could be definitively diagnosed from the histopathological findings, hepatic steatosis could not be identified by preoperative imaging modalities such as abdominal ultrasonography or dynamic CT, and for whom the food history and occupational history were unknown, were considered to be patients with no obvious etiology or patients with cryptogenic HCC.

Among NBNC-HCC patients, 29 (14.9%) had HCC associated with NASH (NASH-HCC), 57 (29.3%) had HCC associated with ALD (ALD-HCC), and 108 (55.8%) had HCC associated with cryptogenic etiology (cryptogenic HCC).

The median age of the patients with cryptogenic HCC was significantly older than the patients with NASH-HCC (*P* = 0.018). The percentages of patients with ALD-HCC who were male or had cryptogenic HCC were significantly higher than the percentage of patients with NASH-HCC (*P* < 0.001).

The median platelet count of patients with cryptogenic HCC was significantly higher than the counts of the other groups of patients (vs. NASH-HCC, *P* = 0.025; vs. ALD-HCC, *P* = 0.023). The FIB-4 index of the patients with cryptogenic HCC was significantly lower than the FIB-4 index of the other groups of patients (vs. NASH-HCC, *P* < 0.001, vs. ALD-HCC, *P* = 0.002). Differences between the other clinical characteristics of the 3 groups of patients were not significant (Table 3).

**Table 3:** Clinical characteristics of patients with NBNC-HCC who underwent hepatic resection

	NASH	Alcoholic liver disease	Cryptogenic etiology	*P* value		
				NASH versus Alcoholic liver disease	NASH versus cryptogenic etiology	Alcoholic liver disease versus cryptogenic etiology
	n = 29	n = 57	n = 108			
Age (years)	66 (47–82)	71 (54–88)	73 (25–85)	0.040	0.018	N.S
Sex (male/female)	14/15	55/2	84/21	< 0.001	< 0.001	0.004
Body mass index (kg/m^2^)	24.4 (17.4–31.3)	23.6 (15.2–32.4)	23.5 (16.0–34.2)	NS	NS	NS
Antibody to hepatitis B core antigen	10 (34.4%)	17 (29.8%)	34 (31.4%)	NS	NS	NS
Diabetes mellitus	13 (44.8%)	30 (54.3%)	64 (59.2%)	NS	NS	NS
Hypertension	17 (58.6%)	34 (59.6%)	71 (65.7%)	NS	NS	NS
Hyperlipidemia	8 (25.5%)	15 (26.3%)	36 (33.3%)	NS	NS	NS
AST (IU/L)	53 (19–397)	56 (19–146)	39 (15–308)	NS	0.041	0.035
ALT (IU/L)	58 (17–435)	45 (11–175)	38 (8–340)	0.035	0.032	0.043
γ-GTP (IU/L)	74 (21–612)	82 (18–836)	70 (8–583)	NS	NS	NS
Platelet counts (× 10^4^/μL)	15.4 (7–23.6)	15.1 (5.2–29.1)	18.4 (6.1–43.3)	NS	0.025	0.023
FIB-4 index	3.68 (1.03–17.52)	3.28 (1.40–11.37)	2.56 (0.47–8.66)	NS	< 0.001	0.002
ALBI grade 1/2/3	17/9/3	38/17/2	74/32/2	NS	NS	NS

Continuous data are presented as medians and range, and categorical data are presented as numbers of patients

*AST* aspartate aminotransferase, *ALT* alanine aminotransferase; *γ-GTP* γ-Glutamytranspeptidase, *NASH* non-alcoholic steatohepatitis, *FIB-4* fibrosis-4, *ALBI grade* albumin-bilirubin grade, *NS* not significant

**Fig. 3:**
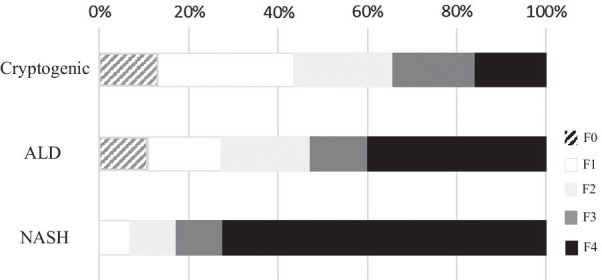
The proportion of patients with NBNC-HCC who underwent hepatic resection according to stages of fibrosis as assessed in nonmalignant regions of resected liver.  : F0;  : F1;  : F2;  : F3;  : F4

The proportions of patients with NASH-HCC with fibrosis stages 0, 1, 2, 3, and 4 were 0%, 6.8%, 10.4%, 10.4%, respectively. The proportions of patients with ALD-HCC with fibrosis stages 0, 1, 2, 3, and 4 were 10.5%, 15.8%, 19.2%, 12.2%, and 38.6%, respectively. The proportions of patients with cryptogenic-HCC with fibrosis stages 0, 1, 2, 3, and 4 were 12.9%, 30.5%, 22.2%, 18.5%, and 15.7%, respectively. Cirrhosis was found in 72.4%, 38.6%, and 15.5% of patients with NASH-HCC, ALD-HCC and cryptogenic HCC, respectively. The prevalence of cirrhosis in patients with NASH-HCC was significantly higher than the prevalences in the other groups of patients undergoing hepatic resection (*P* < 0.001) (Fig. 3).

## Discussion

We retrospectively assessed the clinical characteristics of patients with HCC in this study. From 1992 to 2018, 514 (23.6%) of 2171 patients had NBNC-HCC. The incidence and the number of patients with NBNC-HCC tended to increase, whereas the incidence and the number of patients with HCV–HCC tended to decrease, especially in recent years. Patients with NBNC-HCC were significantly older, and the rates of excessive alcohol consumption, T2DM, hypertension, and hyperlipidemia were significantly higher than those rates in the other groups of patients. Furthermore, in patients with NBNC-HCC, the BMI and platelet count were significantly higher and the FIB-4 index was significantly lower than those values in the other groups of patients. Previous studies have also reported that patients with obesity, T2DM, excessive alcohol consumption, and metabolic syndrome are at an increased risk of developing HCC [45, 46]. Therefore, as we previously reported [8], these complications are likely to become risks for future HCC and need attention.

As shown in Table 2, the patients identified with NBNC-HCC from 2010 to 2018 were significantly older, and the rates of T2DM, hypertension, and hyperlipidemia were higher than in the patients identified with NBNC-HCC from 1992 to 2009. We think that the increased incidence of metabolic syndrome might be associated with the increased incidence of NBNC-HCC. On the other hand, the platelet count was higher and the FIB-4 index was lower in patients with NBNC-HCC from 2010 to 2018 than in the earlier patients from 1992 to 2009. These results might be explained by the fact that HCC occurred even in patients with non-advanced liver fibrosis than before. In general, liver fibrosis is a progressive condition, and HCC develops from cirrhosis. Moreover, the current EASL guidelines require a biopsy for patients with HCC who do not have cirrhosis [47]. However, predicting the development of HCC in our patients whose fibrosis had not progressed was difficult. Therefore, as shown in Table 2, even in some of the patients seen from 2010 to 2018, a FIB-4 index of ≥ 3.25 indicated severe fibrosis (F3–F4) [48]. In Japan, the recommendation has been to use the FIB-4 index as one of the methods for screening patients who have been identified with possible advanced liver fibrosis [49]. Furthermore, the FIB-4 index tended to be higher in the older study patients in general; but as shown in Fig. 2, the differences between patients stratified by age were not significant. On the other hand, although in patients with HCC, the metabolic syndrome might be associated with a systemic inflammatory condition, which might be associated with thrombocytosis, the reason why more platelets were maintained in patients with HCC from 2010 to 2018 than in patients from 1992 to 2009 has not been clarified in this study. Additional studies are needed.

The TNM stages of HCC in patients with NBNC-HCC were significantly more advanced than the stages of the other groups of patients. HBV and HCV infection are well known to be chronic, and the progression of liver fibrosis often leads to chronic hepatitis, cirrhosis, cirrhosis-related complications, and HCC [50]. Our patients with HBV and HCV therefore underwent regular surveillance. In addition, a large prospective study [51] observed a lower rate of hepatic decompensation and mortality in patients with cirrhosis associated with NASH than in patients with cirrhosis associated with HCV.

However, for patients who develop NBNC-HCC, the established screening method has remained unclear. If there is a high possibility that liver fibrosis will progress, it is important to perform screening by CT and MRI and apply a new imaging and scoring system. We think that such screening and scoring will allow the earlier diagnosis of HCC [52–54].

Histopathological examinations of nonmalignant hepatic tissue from patients with NASH-HCC, ALD-HCC, and cryptogenic HCC identified cirrhosis in 72.4%, 38.6%, and 15.5% of patients, respectively. The prevalence of cirrhosis in patients with NASH was significantly higher than the prevalence of cirrhosis in other groups, and patients with cryptogenic HCC had the lowest rate of advanced fibrosis. Many study reports have shown that NASH can progress to cirrhosis [55–58], and the authors concluded that the severity of liver fibrosis must be estimated to determine the surveillance and for treatment of NAFLD. In this study, the patients with cryptogenic HCC tended to have elevated platelet counts, a decreased FIB-4 index, and nonsevere fibrosis. On the other hand, resected hepatic tissue samples are biased, since the samples likely included more patients without cirrhosis, who are more likely to undergo hepatic resection because they are more likely to have adequate liver function. However, based on the results of our histopathological diagnoses, we concluded that HCC can develop in noncirrhotic patients.

In this study, the median FIB-4 index of the patients with cryptogenic HCC was 2.56. At present, the FIB-4 index, a scoring system that has been recommended along with other systems, uses a cutoff value of 2.67 to indicate that fibrosis might progress. Therefore, this value is similar to the value that has been regarded as an index that increases the risk of developing HCC [59, 60]. Thus, the FIB-4 index may also be useful for screening patients for the risk of HCC.

Our study identified many patients with NBNC-HCC who had HCC with a cryptogenic etiology. Most (93.5%) of the patients with cryptogenic-HCC had any of obesity, T2DM, hypertension, hyperlipidemia, and HBcAb. Taken together, these patients with NBNC-HCC who had HCC with a cryptogenic etiology might have progressed without being found to have NASH or ALD during their follow-ups. In the future, physicians should monitor patients with metabolic syndrome for the development of HCC.

In this study, the incidence and the number of patients with NBNC-HCC tended to increase during the most recent study period, from 2010 to 2018. In addition, many of these patients often had T2DM, obesity, and metabolic syndrome. Furthermore, while some of these patients could be identified to have NASH from a histopathological examination, a definitive histopathological diagnosis was not always possible. Furthermore, we did not necessarily show cirrhosis in histologically. However, although it is certain that HCC surveillance in patients with NBNC-HCC without cirrhosis has not yet been established, the FIB-4 index of patients with NBNC-HCC without cirrhosis tended to be higher. Therefore, we concluded that the FIB-4 index might become a useful screening method.

This study has limitations. Not all the histopathological aspects of background liver were available in the NBNC-HCC group. Our study patients may not reflect all of the patients with HCC in Japan. We hope to assess the association between the FIB-4 index and the risk of HCC in the cohort study.

## Conclusions

The incidence of NBNC-HCC has gradually been increasing. Most recently, 40% of all patients with HCC had NBNC-HCC. Metabolic syndrome was an important risk factor for NBNC-HCC, and HCC was not uncommon in patients who did not have cirrhosis. However, the FIB-4 index tended to be higher and could be a useful screening method for HCC.

## Data Availability

The datasets used and/or analysed during the current study available from the corresponding author on reasonable request.

## Abbreviations

HCC: Hepatocellular carcinoma
HBV: Hepatitis B virus
HCV: Hepatitis C virus
NASH: Non-alcoholic steatohepatitis
ALD: Alcoholic liver disease
